# Compound Comminuted Fracture of the Frontal Bone—Trephining—Recovery

**Published:** 1881-02

**Authors:** James S. Smith


### COMPOUND COMMINUTED FRACTURE OF THE FRONTAL BONE—TREPHINING—RECOVERY.

REPORTED BY JAMES S. SMITH, M. D.

G. R., aged 21 years, was struck upon the head on October 9, 1880, with a fence picket, and knocked down. He lay insensible for a time, but revived and was able to walk home without aid and retired to bed. He awakened his family in the morning by his moaning, and was found covered with blood and partly insensible. I was called October 10, in the morning, and found a punctured wound, about half an inch long, and a fracture of the frontal bone on the left side, near the temporal bone. The fracture was star-shaped, and with one of the triangular pieces considerably depressed. He was semi-conscious, restless, could not answer questions correctly, called everybody by his own name, but did not complain of any pain. His pulse was 50, full, respirations irregular. Dr. Diehl was called in consultation and advised non-interference, but to carefully watch the patient. His condition remained about the same until next morning (Oct. 11), when he became more restless, with more marked symptoms of depression. The pulse continued slow, and the temperature was slightly elevated (101). Dr. Mynter was called in the afternoon, and advised immediate trephining, and removal of the depressed piece of bone. The operation was performed by a cross-shaped incision, the periosteum was lifted up by elevator, and a triangular depressed piece of bone, about ¾ inch long in all directions, removed by Hay's saw. The internal plate ot the bone was considerably larger in extent than the external. Under the depressed piece of bone a large blood coagulum was found and partly removed; dura mater seemed intact.

The pulse immediately assumed its normal condition and all the symptoms of pressure gradually disappeared. The wound was partly sutured and covered with carbolized water solution. The farther progress was favorable, and in five weeks the wound was completely healed and the patient discharged cured.

